# The fate of biodegradable polylactic acid microplastics in maize: impacts on cellular ion fluxes and plant growth

**DOI:** 10.3389/fpls.2025.1544298

**Published:** 2025-02-25

**Authors:** Shijia Bao, Xi Wang, Jianxiong Zeng, Le Yue, Zhenggao Xiao, Feiran Chen, Zhenyu Wang

**Affiliations:** ^1^ Institute of Environmental Processes and Pollution Control, and School of Environment and Ecology, Jiangnan University, Wuxi, China; ^2^ College of Forestry and Grassland, Nanjing Forestry University, Nanjing, China

**Keywords:** biodegradable microplastics, polylactic acid, transformation, distribution, biological effects

## Abstract

The widespread application of biodegradable microplastics (MPs) in recent years has resulted in a significant increase in their accumulation in the environment, posing potential threats to ecosystems. Thus, it is imperative to evaluate the distribution and transformation of biodegradable MPs in crops due to the utilization of wastewater containing MPs for irrigation and plastic films, which have led to a rising concentration of biodegradable MPs in agricultural soils. The present study analyzed the uptake and transformation of polylactic acid (PLA) MPs in maize. Seed germination and hydroponic experiments were conducted over a period of 5 to 20 days, during which the plants were exposed to PLA MPs at concentrations of 0, 1, 10, and 100 mg L^-1^. Low concentrations of PLA MPs (1 mg L^-1^ and 10 mg L^-1^) significantly enhanced maize seed germination rate by 52.6%, increased plant shoot height by 16.6% and 16.9%, respectively, as well as elevated aboveground biomass dry weight by 133.7% and 53.3%, respectively. Importantly, depolymerization of PLA MPs was observed in the nutrient solution, resulting in the formation of small-sized PLA MPs (< 2 μm). Interestingly, further transformation occurred within the xylem sap and apoplast fluid (after 12 h) with a transformation rate reaching 13.1% and 27.2%, respectively. The enhanced plant growth could be attributed to the increase in dissolved organic carbon resulting from the depolymerization of PLA MPs. Additionally, the transformation of PLA MPs mediated pH and increase in K^+^ flux (57.2%, 72 h), leading to acidification of the cell wall and subsequent cell expansion. Our findings provide evidence regarding the fate of PLA MPs in plants and their interactions with plants, thereby enhancing our understanding of the potential impacts associated with biodegradable plastics.

## Graphical Abstract

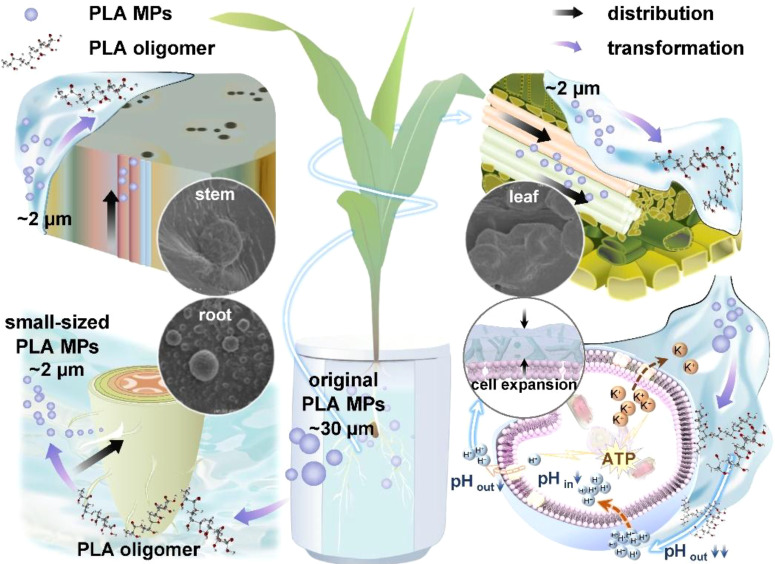

## 1 Introduction

The emergence of plastics has revolutionized numerous industries (Gu et al., 2020). However, the accumulation of plastic waste, especially in the form of microplastics (MPs) defined as plastic fragments smaller than 5 mm, poses significant environmental challenges (Xia et al., 2023; Thompson et al., 2004). Polylactic acid (PLA), as the most widely used biodegradable plastic, accounts for over 30% of global biodegradable plastic production capacity (Maximize Market Research, 2022). Compared to conventional non-biodegradable plastics, PLA plastics exhibits a higher tendency to degrade into smaller particles (Song et al., 2022). For example, PLA MPs have been observed to form within a 12-week period under warm and illuminated conditions (Apicella et al., 2024). Wastewater contains MPs derived from plastic waste, synthetic fibers, and microbeads (Hu et al., 2024). When utilized for irrigation, these MPs are introduced into agricultural soils. Additionally, sewage sludge, commonly applied as fertilizer due to its nutrient-rich composition, also serves as a notable source of MPs (Hechmi et al., 2024). Consequently, the application of sewage sludge as fertilizer, along with the degradation of plastic mulching films, has transformed agricultural systems into substantial reservoirs for PLA MPs (Mahon et al., 2017; Maity et al., 2022; Serrano-Ruiz et al., 2021; Jamil et al., 2024). For example, concentrations exceeding 1 mg kg^-1^ of PLA MPs have been detected in various agricultural soils, including vegetable farms and rice paddies (Liu et al., 2018; Wang et al., 2022). The presence of MPs in the environment can be absorbed by plants and accumulate in their edible parts, thereby influencing plant growth and biomass (Li et al., 2020). However, current understanding regarding the uptake of PLA MPs by plants and its resulting effects remains limited. Therefore, investigating the transformation and distribution of PLA MPs within plant-associated systems will enhance our understanding of the biological effects of PLA materials, thereby improving management practices for mitigating MP impacts in agriculture.

Current studies on PLA MPs have primarily focused on comparing their effects with conventional non-degradable MPs, specifically examining the impact on seed germination, biomass, and photosynthesis of plants such as maize (*Zea mays* L.), tomatoes (*Solanum lycopersicum* L.), rice (*Oryza sativa* L.), soybean (*Glycine max* (L.) Merr.) and oat (*Avena sativa* L.) (Chu et al., 2023; Yang et al., 2021; Yu et al., 2023). Low concentrations (*e.g*., 2.5%, w/w) of PLA MPs in soil did not exhibit any detrimental effect on the seed germination rate (with an average rate no lower than 88.9%) of *Sorghum saccharatum* and *Lepidium sativum* (Liwarska-Bizukojc, 2022). Similarly, exposure to soil containing PLA MPs ranging from 20 to 60 µm at a concentration of 0.1% (w/w) did not affect the fresh weight of maize shoots (Lian et al., 2022). Soybean (*Glycine max* (L.) Merr.) and oat (*Avena sativa* L.) exposed to PLA MPs at a concentration of 0.2% (w/w) also showed no significant effects on roots or yields (Chu et al., 2023). Generally, low concentrations of PLA MPs appear to be non-toxic to plants; however high concentrations may pose certain hazards (Liu et al., 2023). It is worth noting that depolymerization can result in the release of PLA oligomers from PLA MPs which can subsequently self-assemble into PLA nanoparticles (Qi et al., 2017; Wang M. et al., 2023). Larger MPs (~200 nm, PS MPs) are known to impede cell wall permeability thereby restricting nutrient uptake in plants (Zhang et al., 2023); whereas smaller MPs (~100 nm, PS MPs) and nanoparticles can penetrate cell walls and membranes affecting antioxidative systems and chlorophyll synthesis (Zhang et al., 2023). Importantly, the biological effects exerted by MPs are closely related to their fate within plants (Jamil et al., 2024, 2025; Zhang et al., 2023, 2022). While previous research has mainly focused on the effects caused by PLA MPs; little is known about their internalization and transformation within plant tissues. Recent studies have indicated that submicrometer or nanoscale polystyrene (PS) plastic particles can enter plants through root cracks via apoplast transport (crack model), followed by internalization into cells through endocytosis (Huang et al., 2022; Li et al., 2020; Luo et al., 2022; Yang et al., 2024). However, it is currently unknown whether PLA MPs will undergo transformation in plant-associated systems and how this process may impact the distribution and phyto-effects of PLA MPs.

In this study, we hypothesize that PLA MPs may undergo depolymerization to form smaller PLA MPs. Subsequently, the resulting MPs can be further internalized into plants through root cracks and undergo further transformation within plant tissues. To investigate the fate of PLA MPs, maize was selected as the target plant due to its global significance as a food source and the extensive use of biodegradable plastic films in its cultivation (Erenstein et al., 2022), indicating an increasing risk of accumulating PLA MPs. The objectives of this study were: (1) to investigate the biological effects of PLA MPs on maize during germination and seedling growth; (2) to examine the uptake of PLA MPs by maize, evaluate the levels of transformation of PLA MPs within plants; and (3) to explore the mechanisms underlying the effects of PLA MPs on plant cell growth. The findings on the transformation and distribution of PLA MPs will contribute to a deeper understanding of potential health risks associated with biodegradable plastics and provide insights into ecological implications to their applications in agroecosystems.

## 2 Materials and methods

### 2.1 Measurements of seed germination indexes after exposure to PLA MPs

PLA MPs (average diameter = 33.32 ± 13.66 μm, molecular weight: 263976, **Supplementary Figures S1**; **S2**) were purchased from Tesulang Chemical Materials Co., Ltd. (Dongguan, China). Maize seeds (*Zea mays* L. cv. Dafeng No. 30) were obtained from Yixin Seed Industry and sterilized by immersion in a 0.5% sodium hypochlorite (NaClO) solution for 30 min followed by rinsing with sterile deionized water to remove residual NaClO. Sterilized intact maize seeds were randomly immersed in PLA MPs suspensions at final concentrations of 0, 1, 10, and 100 mg L^-1^ for two hours at room temperature. The selected concentrations of PLA MPs were based on the average PLA MP concentration found in agricultural soils (1 mg kg^-1^) and referenced from concentrations used in studies regarding PLA MPs (0–2000 mg kg^-1^) (Chu et al., 2023; Liu et al., 2018). Subsequently, eight soaked maize seeds (8 technical replicates) were evenly placed on qualitative filter paper-lined Petri dish (90 mm diameter), each containing 5 mL of the respective suspension before being incubated in darkness at 28°C for seven days with five replicates per treatment (5 biological replicates). Germination rate (GR), germination vigor (GV), germination index (GI), vigor index (VI), and mean germinating time (MGT) were measured when bud length surpassed half of seed length (see calculation details in **Supplementary Text S1**) (Miransari and Smith, 2014). Additionally, the seed water absorption rate was calculated based on weight change before and after water uptake over the course of seven days (Li et al., 2022).

### 2.2 Plant culture and determination of PLA MP distribution in maize seedlings

To investigate the uptake and distribution of PLA MPs, hydroponic culture was conducted to eliminate the interference of soil components. Maize seedlings were germinated after surface sterilization, and uniform seedlings were selected and transferred into ceramic hydroponic tanks spiked with half-strength modified Hoagland solution (**Supplementary Table S1**) (Wang et al., 2012). After one week of acclimation (25°C, 14/10 h light/dark cycle), four treatment groups were initiated in full-strength Hoagland nutrient solution containing 0 (control), 1, 10, and 100 mg L^-1^ PLA MPs, respectively. Eight jars were set up for each treatment. Shoot and root tissues were harvested at 5, 10, 15, and 20 d after exposure. The samples were washed with tap water followed by three rinses with deionized water. The roots were scanned and the morphological parameters were analyzed using WinRHIZO Pro 2005 b (Regent Instruments Inc., Canada). Fresh and dry weight measurements were determined accordingly (Wang et al., 2012).

To visualize the distribution of PLA MPs in roots, stems, and fully expanded leaves near the primary veins, scanning electron microscope (SEM, S-4800 field emission scanning electron microscope, Hitachi, Japan) was used for mapping samples exposed to PLA MPs (100 mg L^-1^). The samples were prepared as previously described (Li et al., 2020). Briefly, samples were sectioned into small pieces followed by freezing in liquid nitrogen. After freeze-drying and coating with gold for 60s (~a thickness of 1 nm), the samples were examined using a SEM. Cross sections were observed at an accelerating voltage of 15 kV in high vacuum mode with backscatter detection, and at least three plants from each treatment were examined.

### 2.3 Measurement of dissolved organic carbon and characterization of PLA MPs in nutrient solution

The changes in DOC in the hydroponic solution are a result of depolymerization of PLA MPs and excretion of root exudates (Cai et al., 2024; Siddiqui et al., 2020). To determine the DOC levels and characterize PLA MPs, samples of hydroponic nutrient solution with and without maize cultivation were collected at intervals of 5, 10, 15, and 20 d under the exposure of PLA MPs (~33.32 μm) at different concentrations (0, 1, 10, 100 mg L^-1^). Root exudates were collected on day 20 using a method as previously described (Zhu et al., 2009). Briefly, three maize seedlings were rinsed with deionized water for three times, and their roots were soaked in sterilized deionized water (50 mL) for 24 h at room temperature. The nutrient solution and root exudate samples were then filtered through a microporous film with a pore size of 0.45 µm followed by measurements using a Total Organic Carbon Analyzer (vario TOC select, Elementar, Germany).

The depolymerization of PLA MPs can be evaluated by analyzing their molecular weight distribution as well as the release of smaller MPs from PLA MPs (Virág et al., 2023). PLA MPs were filtered from the aforementioned nutrient solutions incubated with or without plants for 20 days. After washing with SDS (1%), distilled water, and ethanol, the samples were dried in a desiccator containing silica gel (Wang M. et al., 2023). The molecular weight distribution analysis was performed on PLA MPs present in the nutrient solution using gel permeation chromatography (GPC), while nanoparticle tracking analysis (NTA) was used to evaluate the release of small-sized MPs (< 2 μm). GPC analysis was conducted at 35°C using a protective column connected to a PLgel MIXED-B LS (300 × 7.5 mm). NTA measurements were carried out using NanoSight NS300 (Malvern Instruments, Malvern, UK) equipped with a laser emitting light at wavelength of 488 nm.

### 2.4 Extraction of xylem sap and apoplast fluid

The 20-day treated plants were collected for extraction of xylem sap and apoplast fluid. Xylem sap of maize was collected following a previously described procedure (Cai et al., 2020). The detailed operational procedures are provided in **Supplementary Text S2**, and the device used for maize xylem sap extraction is shown in **Supplementary Figure S3A**. The apoplast fluid was extracted based on a published protocol with slight modifications (Gentzel et al., 2019). In brief, the extraction targeted the apical meristem of maize seedlings specifically focusing on the first true leaf tip. Due to their small size, each sample yielded approximately a 4 cm long leaf tip that underwent meticulous processing to extract its apoplast contents (**Supplementary Text S3**; **Supplementary Figure S3B**).

### 2.5 Incubation of PLA MPs with extracted sap and characterization of PLA MP transformation

The transformation of PLA MPs was assessed by applying fluorescein isothiocyanate (FITC)-labelled PLA MPs (purchased from Shanghai Sur-Release Biotech Inc., China) to measure the fluorescence quenching level. FITC-labelled PLA MPs (1 mg L^-1^) were incubated with deionized water, xylem sap, and apoplast fluid for 24 h, respectively. The structure of FITC-labelled PLA MPs was determined using ^1^H-, and ^13^C nuclear magnetic resonance (NMR) Spectrometer (Bruker Avance III, 400 MHz) at room temperature with deuterated chloroform as the solvent (**Supplementary Figure S4**). Furthermore, the fluorescence of FITC-labelled PLA MPs after incubation was observed using a fluorescence microscope (Nikon Ni-U, Japan), and Image J (v1.8.0) software was utilized to measure the average fluorescence intensity.

### 2.6 Cell culture in the presence of PLA MPs

To further investigate the effects and mechanisms of PLA MPs on plants at the cellular level, Bright Yellow-2 (BY-2) tobacco (*Nicotiana tabacum* L.) cells were utilized as a representative plant cell system (Brandizzi et al., 2003). The BY-2 cells (provided by the College of Life Science, Shandong Agricultural University, China) were incubated in full-strength Murashige & Skoog (MS) culture medium under dark conditions with agitation at 130 rpm at 24°C. The BY-2 cells (40 g L^-1^ each) were challenged with PLA MPs (0, 0.01, 0.1, 1, 10, and 100 mg L^-1^) during the exponential growth stage in half-strength MS medium. Considering the preliminary results related to cell viability (3 independent experiments, 3 replicates for each experiment) and both fresh and dry weight (experimental details provided in **Supplementary Text S4**), as well as accounting for cumulative effects during environmental exposure, 0.1, 1, and 100 mg L^-1^ were chosen as the concentrations of interest for assessing subsequent cellular responses.

### 2.7 Evaluation of cellular responses to PLA MPs

Fluorescein diacetate (FDA)/propidium iodide (PI) double staining was employed to visualize the viability of cells exposed to PLA MPs at concentrations of 1, 10, and 100 mg L^-1^ after 12 and 72 h, following previously described procedures (Poborilova et al., 2013). Briefly, 100 μL cell suspensions were incubated with 5 mg L^-1^ FDA and 20 mg L^-1^ PI for 5 min. The cell viability was assessed by quantifying the percentage of viable (FDA positive) and dead (PI positive) cells within the field of view using a fluorescence microscope (Nikon Ni-U, Japan).

Net fluxes of H^+^ and K^+^ were measured using Non-invasive Micro-test Technology (NMT, NMT100S-SIM-XY, Xuyue, Beijing, China). Cells were collected from the control, 0.1, 1, and 100 mg L^-1^ PLA MPs treatments at different time intervals. One mL cell suspension was naturally sedimented in a funnel made from a membrane with pore size of 0.45 μm; over time cells became enriched at the tip region inside the funnel. Ion fluxes were measured approximately between distances ranging from about 1-2 μm away from the surface area surrounding each cell (details provided in **Supplementary Text S5**).

The intracellular pH (pH_in_) was measured using a pH_in_ detection kit (Beijing Solarbio Science & Technology Co., Ltd). 2’,7’-bis(2-carboxyethyl)-5,6-carboxyfluorescein acetoxymethyl ester (BCECF-AM) is the most commonly utilized fluorescent probe for detecting intracellular pH (Wollenburg et al., 2021). Cells were suspended in HEPES buffer solution at a concentration of 4 × 10^7^ cells mL^-1^, followed by adding 1 mM BCECF-AM/dimethyl sulfoxide (DMSO) solution (the solution obtained by dissolving BCECF-AM in DMSO) to reach a final BCECF-AM concentration of 3 μM. The mixture was incubated at 27°C for 30 min. Subsequently, the cells were washed with HEPES buffer for three times and resuspended to a concentration of 3 × 10^6^ cells mL^-1^. The fluorescence intensity of cells was measured using a microplate reader (Thermo Scientific, USA). A calibration curve (**Supplementary Figure S5**) was constructed from the results obtained by incubating BCECF-loaded cells with 50 mM KCI and 200 mg L^-1^ nigericin in buffers at various pH values; nigericin was added to achieve equilibrium between intracellular and extracellular pH levels in the presence of depolarizing concentrations of extracellular K^+^. Extracellular pH (pH_out_) was determined by measuring the pH of the supernatant after cell culture.

An ATP kit (purchased from Beyotime Biotech Inc.) employing a chemiluminescence method was used to determine the intracellular ATP content of BY-2 cells. BY-2 cells were separated from the medium, and their ATP content was measured according to manufacturer’s instructions (Beyotime, China) using a microplate reader (Thermo Scientific, USA). Technical replications were conducted for quality control. The intracellular ATP content was calculated based on the standard curve (**Supplementary Figure S6**).

### 2.8 Statistical analysis

Data are presented as mean ± standard deviation (three replicates per treatment unless otherwise specified). A one-way analysis of variance (ANOVA) followed by a least significant difference (LSD) test was performed to determine the significant differences within treatments. Statistical significance was considered at *p*<0.05.

## 3 Results and discussion

### 3.1 Effects of PLA MPs on seed germination

**Figure 1:**
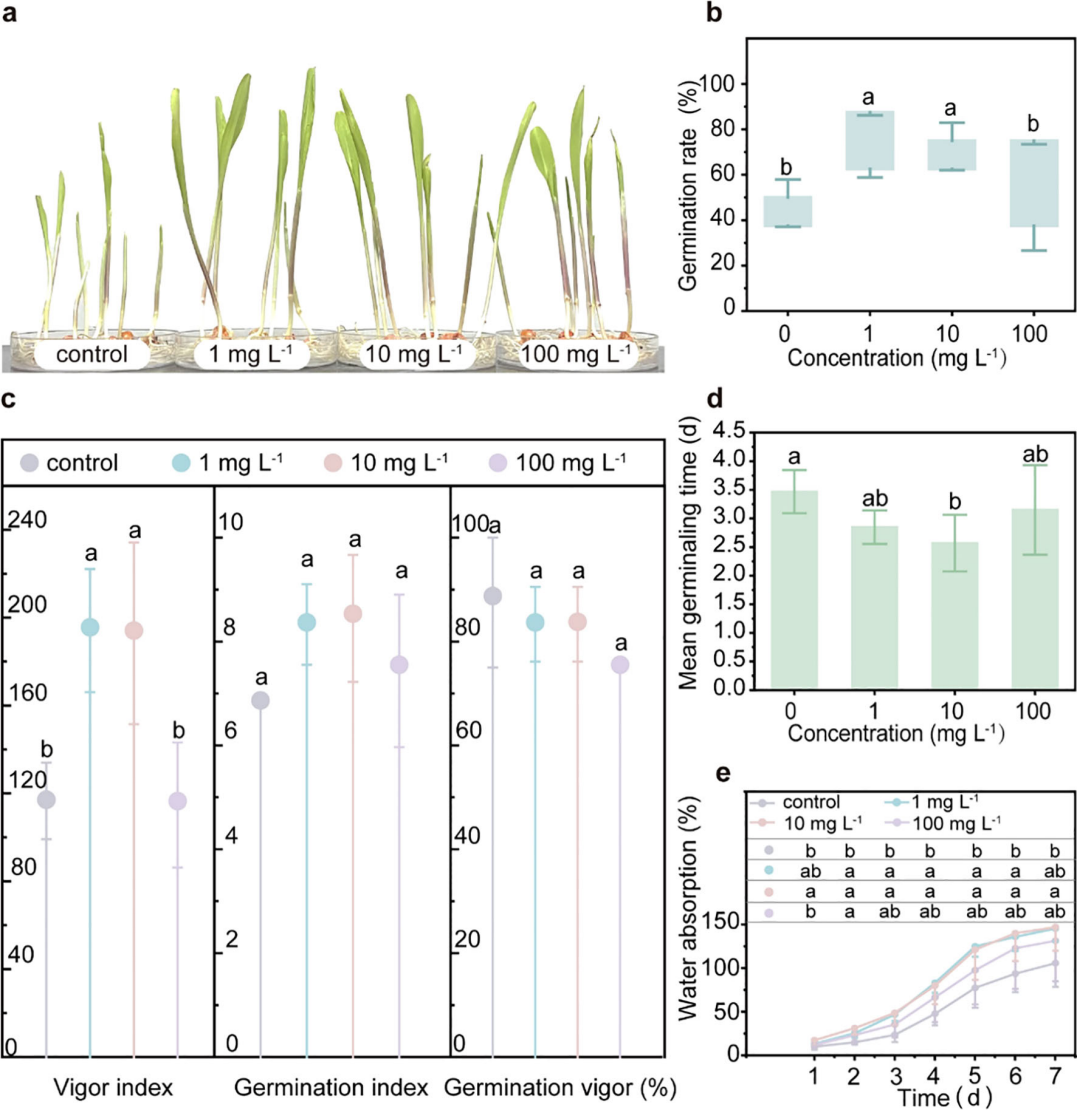
Impacts of PLA MPs on the germination of maize. Photos of maize seedlings after 7-day germination under exposure to PLA MPs (0, 1, 10, and 100 mg L^-1^) **(A)**. Effects of PLA MPs on GR **(B)**, VI, GI, GV **(C)**, and MGT **(D)**. Water absorption of maize seeds **(E)**. Lower case letters represent significant differences among treatments. 5 biological replicates (5 dishes), 8 technical replicates (8 seeds per dish), values are means ± SD (n = 5). ANOVA followed by an LSD test was performed to determine the significant differences within treatments. Statistical significance was considered at *p*<0.05.

During the 7-day germination test, the relatively low concentrations of PLA MPs (1 mg L^-1^ and 10 mg L^-1^) significantly enhanced both the germination rate (seed germination capacity) by 52.6% for both concentrations and the vigor index (growth potential of seedlings) by 66.5% and 65.5%, respectively. Additionally, these concentrations significantly reduced the mean germination time by 0.6 days (from 3.4 days to 2.8 days) and 0.9 days (from 3.4 days to 2.5 days). In contrast, the high concentration (100 mg L^-1^) did not have any effect on these parameters (*p*<0.05, **Figures 1A–D**; **Supplementary Figure S7**). However, there was no impact on the germination index or germination vigor (**Figure 1C**), indicating that seed vigor and uniformity of seed germination remained consistent regardless of exposure to PLA MPs. Water absorption is the main driving force for seed germination (Yang et al., 2022). The presence of low concentrations of PLA MPs (1 and 10 mg L^-1^) significantly enhanced (by 1.7- and 1.9-fold) the water absorption rate in maize seeds (*p*<0.05, **Figure 1E**), suggesting that PLA MPs can regulate water absorption to improve seed germination. However, previous studies have reported that fibrous-shaped PLA MPs (75 μm) obstructed root pores which hindered seed hydration and subsequent germination (Yu et al., 2023). It should be noted that in our study we used round-shaped PLA MPs with an average diameter of 33.32 μm (**Supplementary Figures S1**, **S2**), which are less likely to cause damage to root morphology. Previous studies have shown that exposure to PS and polypropylene (PP) MPs increased the seed germination rate of rice (PS MPs, 200 nm, 0.1 mg L^-1^ and 10 mg L^-1^) and cherry tomato (by 111.11%, PP MPs, <500 μm, 10 g L^-1^), the promoting effects were attributed to the enhanced α-amylase activity facilitating starch hydrolysis into monosaccharides (Shorobi et al., 2023; Zhang et al., 2021). Besides, some studies indicate that MPs (PP MPs, <500 μm) may enhance water retention around seeds or modify seed surface properties (such as roughness and morphology), thereby facilitating water uptake (de Souza MaChado et al., 2018; Wang et al., 2021). Additionally, the presence of PLA MPs can decrease the pH of the germination environment (Liu et al., 2023), thereby facilitating cellular elongation during seed germination (Follmer et al., 2021). It is known that FLOE1 (a water-sensing protein) and OsPIP1;1 (an aquaporin) play crucial roles in promoting water uptake for seed germination (Dorone et al., 2021; He et al., 2022). Thus, future experiments could focus on investigating the impact of PLA MPs on these proteins to enhance hydration and improve germination.

### 3.2 Seedling growth in the presence of PLA MPs

**Figure 2:**
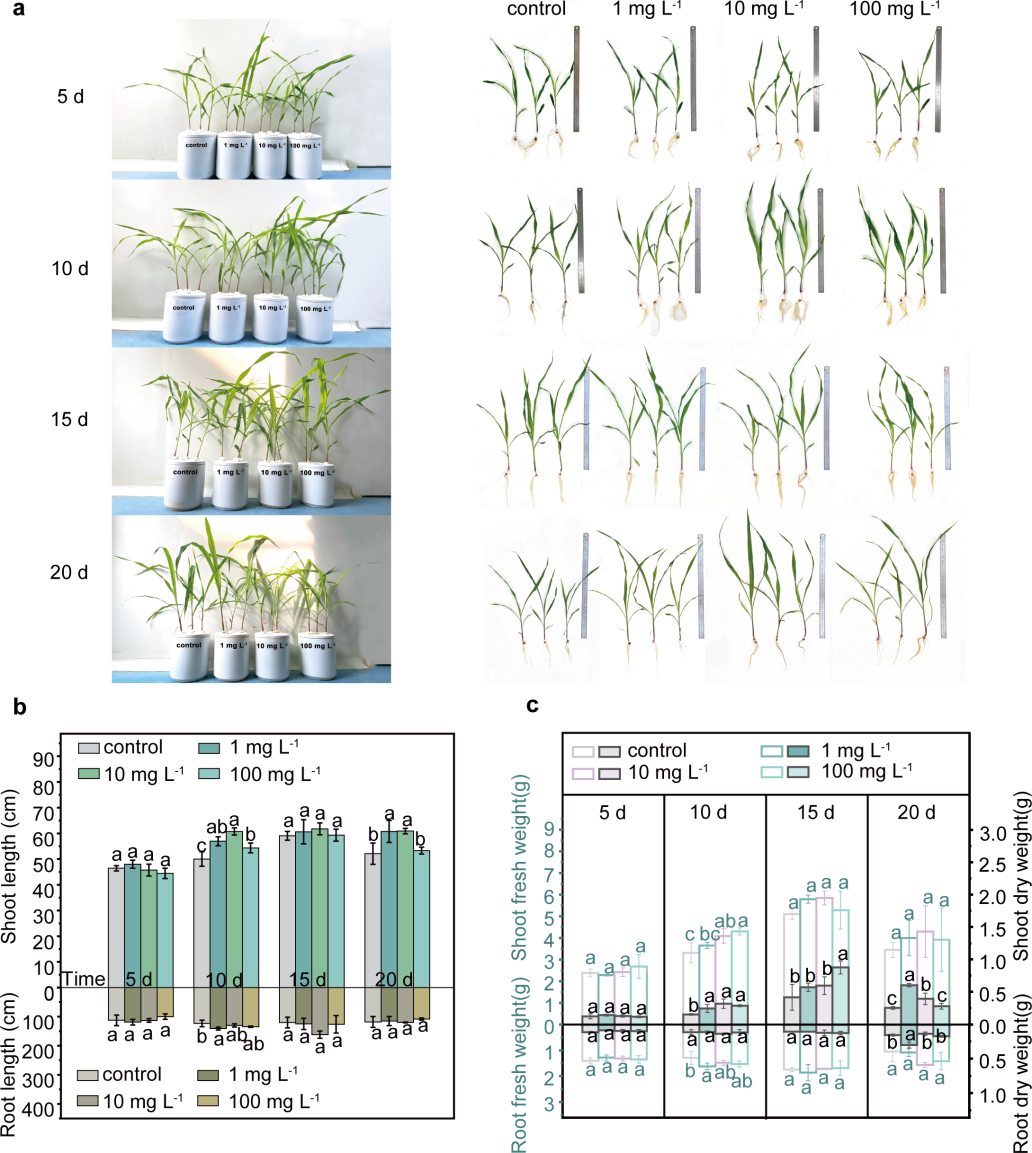
Growth of maize seedlings in the presence of PLA MPs (1, 10, and 100 mg L^-1^). Photos of hydroponic culture and seedlings during 5 to 20 days of incubation **(A)**. Effects of PLA MPs on root/shoot length **(B)**, and fresh (hollow bars) or dry (solid bars) weight of roots or shoots **(C)**. Lower case letters represent significant differences among treatments. 3 biological replicates (3 seedlings), values are means ± SD (n = 3). ANOVA followed by an LSD test was performed to determine the significant differences within treatments. Statistical significance was considered at *p*<0.05.

The root length was similar in both control and PLA MP exposure groups at different concentrations (**Figures 2A, B**). After 20 days of exposure, the average shoot length in the low-concentration groups (1 and 10 mg L^-1^) of PLA MPs showed a significant increase compared to the control group (16.6% and 16.9%, respectively, *p*<0.05), while no significant difference was observed in the presence of 100 mg L^-1^ PLA MPs (**Figure 2B**). Similarly, a significant increase (9.9%-180.1%, *p*<0.05) in dry weight and fresh weight of the shoots was observed after a 10-day exposure to PLA MPs at concentrations of 1, 10, and 100 mg L^-1^, with an additional increase of shoot-fresh weight by 13.3-24.3% after 20-day exposure (**Figure 2C**). Additionally, low rates of PLA MP exposure (0.1% and 1%) were found to enhance maize dry weight in shoots and roots (Yang et al., 2021). This promoting effect may be attributed to the dose-dependent release of certain compounds during degradation of PLA MPs that stimulate plant growth (Tyagi et al., 2018). Furthermore, it has been suggested that PS MPs can enhance seedling growth by promoting plant photosynthesis (Lian et al., 2020). However, the chlorophyll content (SPAD index) and maximum photosystem II quantum yield (Fv/Fm) remained unaffected following PLA MP exposure (**Supplementary Figure S8**). Interestingly, MPs have been demonstrated to affect plant photosynthetic rates and growth by altering soil DOC levels (Ren et al., 2021), and they can also penetrate plant tissues to promote plant growth (Dissanayake et al., 2022). Considering the significant enhancement in shoot growth, further investigation is needed on how PLA MPs influence DOC levels in hydroponic solutions and their distribution pattern within plants.

### 3.3 Alteration of DOC content in the hydroponic solution and distribution of PLA MPs in plants

**Figure 3:**
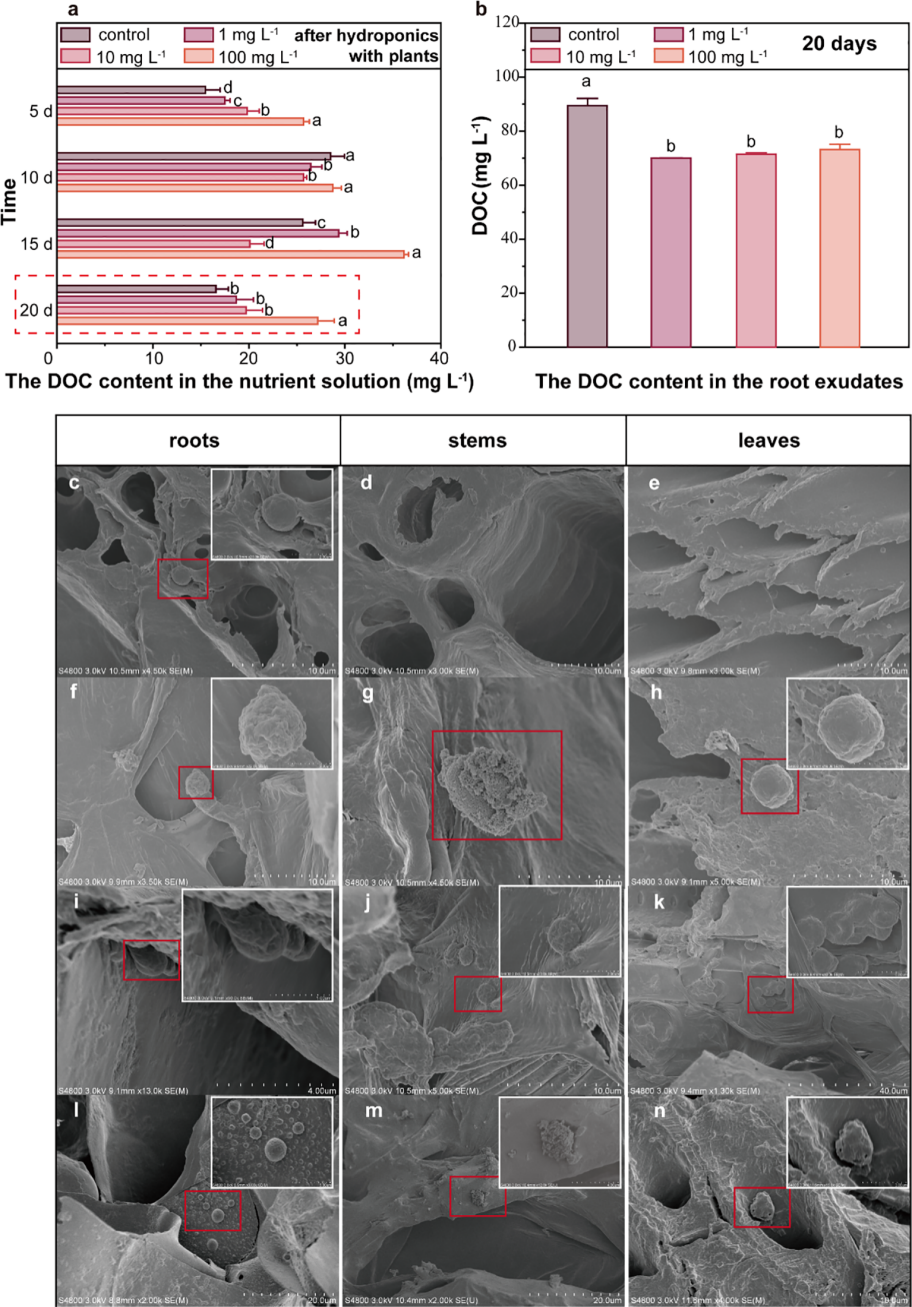
DOC content in the nutrient solution and root exudates, distribution of PLA MPs in plant tissues. The DOC content in the nutrient solution after 5, 10, 15, and 20 days of hydroponic culture, data are represented as means ± SD (n = 3) **(A)**. The DOC content in root exudates after 20-day exposure, data are represented as means ± SD (n = 3) **(B)**. SEM images of PLA MPs observed in the primary and secondary roots of maize **(C, F, I, L)**, stems **(D, G, J, M)** and leaves **(E, H, K, N)** following PLA MP exposure for 5 **(C–E)**, 10 **(F–H)**, 15 **(I–K)**, and 20 **(L–N)** days. Inserts with white frames are magnification of selected area (red frame). ANOVA followed by an LSD test was performed to determine the significant differences within treatments. Statistical significance was considered at *p*<0.05.

Upon exposure to PLA MPs (~33.32 μm, 1, 10, and 100 mg L^-1^), an increase of DOC content in Hoagland nutrient solution collected after hydroponics with plants was observed. Specifically, the DOC level increased by 12.8-64.1% in the presence of PLA MPs after 20 days (1, 10, and 100 mg L^-1^, **Figure 3A**, *p*<0.05). The increase became more pronounced at 100 mg L^-1^ of PLA MPs, while a greater promoting effect on plant growth was observed at relatively low concentrations of PLA MPs (1 and 10 mg L^-1^, **Figures 2B, C**). This inconsistent result may be attributed to the partial inhibition of nutrient uptake due to the deposition of high doses of MPs on root surface (Zhang et al., 2021). The increase of DOC content in the nutrient solution may be attributed to either root exudates or the depolymerization of PLA MPs. Root exudates released from plants can affect the composition of DOC (Lei et al., 2023). To eliminate the effect of exudates on total DOC levels, the DOC content in maize root exudates after 20 days of hydroponic growth was assessed. The DOC concentrations for control and PLA MP treatment groups (1, 10, and 100 mg L^-1^) were 80.9, 64.5, 66.1, and 67.9 mg L^-1^, respectively, demonstrating a significant reduction in DOC levels by approximately 18% compared to the control (**Figure 3B**, *p*<0.05). The regulation of root exudates likely arises from the extensive adsorption of MPs onto root surfaces, thereby interfering with plant metabolic pathways and carbon distribution (Wang B. et al., 2023). Moreover, the increase in total DOC could not be solely attributed to the root exudates. Previous research suggested that PLA MPs may alter the inorganic and organic carbon content in the environment, leading to an increase in algal biomass (Wang et al., 2024). After 20 day-exposure to PLA MPs (~33.32 μm, 1, 10, and 100 mg L^-1^), an increase of DOC content was observed in hydroponic solution without plants, the DOC level increased by 86.3-210.5% (**Supplementary Figure S9**, *p*<0.05). PLA MPs can result in a significant increase in DOC content of water when incubated with deionized water (Sun et al., 2022), which may be related to the depolymerization of PLA MPs to form water-soluble low molecular weight oligomers (Nik Mut et al., 2024). Future research should focus on whether the presence of plants affects the depolymerization of PLA MPs (e.g., through the secretion of root exudates), and explore the particular factors and mechanisms that are implicated.

The PLA MPs were further characterized by conducting SEM scanning of exposed roots, stems, and leaves after incubation (**Figures 3C–N**). Interestingly, the roots exhibited the presence of small-sized PLA MPs (~2 μm) after a 5-day exposure, while no PLA MPs were observed in stem and leaf samples (**Figures 3C–E**). Notably, the PLA MPs appeared on the cell walls of vascular and cortical tissues in both primary and secondary roots (**Figures 3C, I**), indicating their transport across the intercellular space within the apoplast transport system. With longer exposure periods (≥10 d), aggregation of PLA MPs was detected in roots, stems, and leaves; notably, a significant accumulation of MP aggregation was found in the main leaf veins of maize exposed PLA MPs for 15 days (**Figures 3I–K**). Overall, it appears that PLA MPs were transported from roots to shoots over time with the highest concentration observed in leaves after 15-day exposure. The limited presence of small-sized PLA MPs observed in short-term root exposure (5 days) may be attributed to narrow root cracks resulting from plant growth; whereas higher amounts of PLA MPs were detected in stems and leaves due to an extension of root cracks (Li et al., 2020). Considering the original size of PLA MPs (~33.32 μm) and the significant distribution throughout aboveground tissues, the mechanisms for the formation of small-sized PLA MPs remain to be revealed.

### 3.4 Transformation of PLA MPs in hydroponic culture-plant system

**Figure 4:**
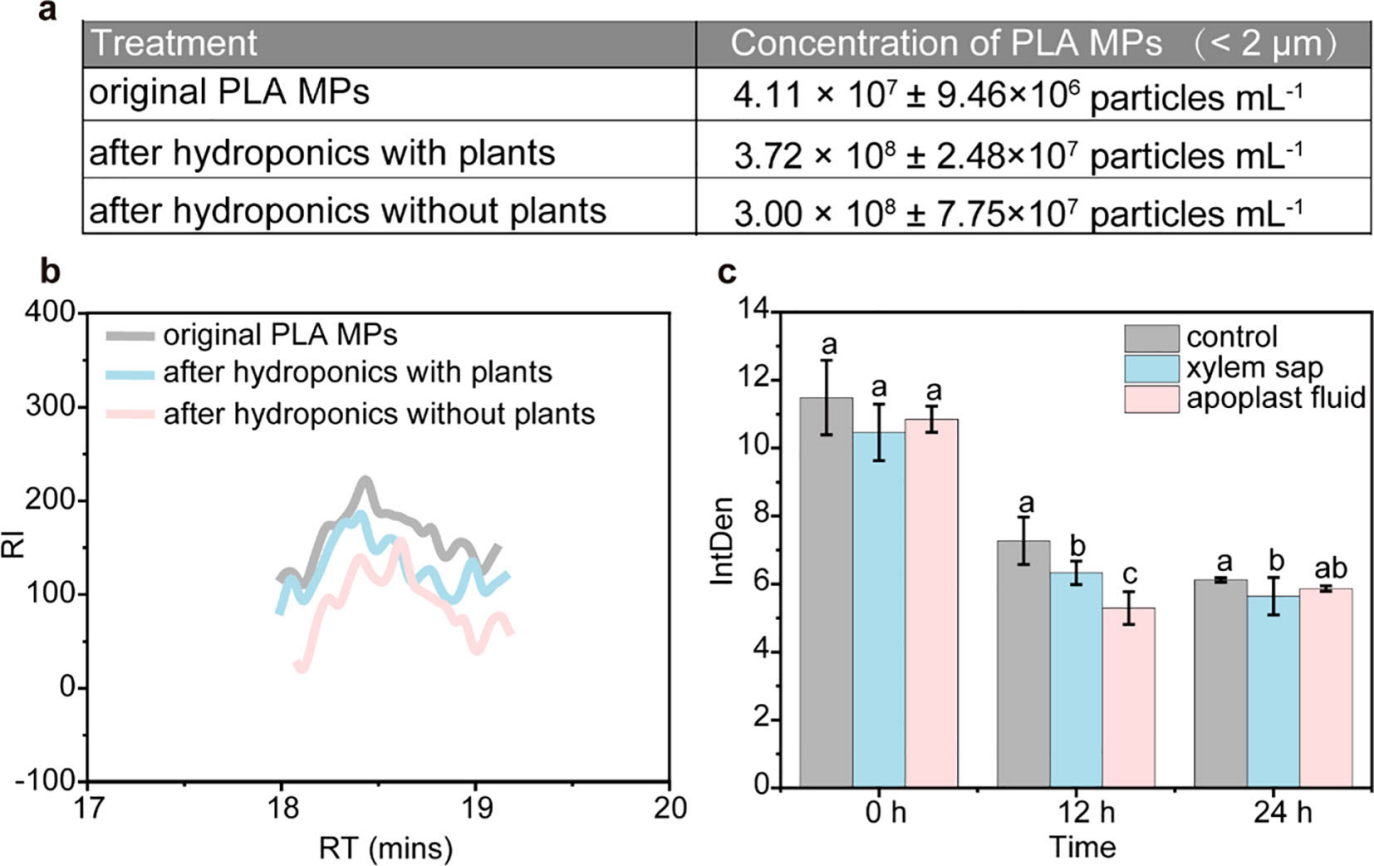
Transformation of PLA MPs in both hydroponic solution and plants. Concentrations of small-sized (< 2 μm) PLA MPs in the nutrient solution as measured by NTA after 20-day exposure **(A)**, data are represented as means ± SD (n = 5). GPC analysis of collected PLA MPs after hydroponic culture with and without plants, compared with the original PLA MP suspensions **(B)**. Mean fluorescence intensity in xylem sap and apoplast fluid after exposure to FITIC-labelled PLA MPs **(C)**, data are represented as means ± SD (n = 6). ANOVA followed by an LSD test was performed to determine the significant differences within treatments. Statistical significance was considered at *p*<0.05.

The transformation potential of PLA MPs necessitates clarification that their transformation occurs prior to absorption by plants. In the hydroponic culture solution, a significant higher amount (by 805.1%, **Figure 4A**) of small-sized (< 2 μm) PLA MPs was detected after 20-day exposure compared to the original PLA MPs. Besides, the amount of small-sized (< 2 μm) PLA MPs remained similar regardless of the presence of plants. It is known that depolymerization of PLA MPs can result in oligomers which can self-assemble into PLA NPs (Wang M. et al., 2023); and PLA may undergo transformation in the environment generating low molecular weight oligomers (Nakayama et al., 2019). Small peaks in the low molecular weight range (~800 g mol^-1^, **Figure 4B**) were detected in the molecular weight distribution of PLA MPs from the nutrient solution. Compared to the original PLA MPs, there was a significant decrease (by 23.9%) in the molecular weight observed in the nutrient solution after 20-day exposure (from 206,202 to 156,912 g mol^-1^, **Supplementary Table S2**), indicating formation of oligomers through depolymerization during hydroponic culture. The depolymerization process for aqueous media is influenced by several key parameters including water absorption degree, macromolecular chain diffusion within polymer matrix, and solubility of resulting degradation compounds (Ainali et al., 2022). Thus, it is likely that small-sized soluble PLA MPs derived from depolymerization could be those detected in roots. However, aggregates formed by PLA MPs observed within vascular tissue (**Figures 3J, M, K**) present different surface morphology than those found in root, suggesting specific transformation within xylem and apoplast fluid.

The fluorescence intensity of xylem or apoplast fluid exposed to FITC-labelled PLA MPs was measured to assess the extent of PLA MP transformation. Following an *in vitro* incubation for 12 h, the average fluorescence intensity decreased significantly by 13.1% and 27.2% in xylem sap and apoplast fluid, respectively (**Figure 4C**). However, extending the incubation time to 24 hours did not result in a further reduction in fluorescence intensity; only a minor decrease of 7.8% (*p*<0.05) was observed in the xylem sap (**Figure 4C**). Considering the high stability of FITC-labelled PLA MPs (**Supplementary Figure S4**), this decline in fluorescence intensity corresponds to the transformation of PLA MPs (Wang M. et al., 2023). The rapid decrease observed within a short incubation time suggests that there may be a fast transformation of PLA MPs occurring in the apoplast fluid. On the other hand, the slower change during longer incubation durations could be attributed to decreased enzyme activity or inhibition by reaction products (Rodríguez-Celma et al., 2016). Apoplast fluid is known for its abundance of enzymes and polysaccharides, while its acidic conditions (pH=5.2-6.5) stimulate specific enzyme activity that accelerates the transformation process of PLA (Geilfus, 2017; Janssens et al., 2023). For instance, extracellular lipases present in apoplast fluid may catalyze ester bonds within PLA MPs, facilitating hydrolysis (Rodríguez-Celma et al., 2016; Wang M. et al., 2023). In contrast, xylem sap primarily consists of water, inorganic ions, and small amounts of organic molecules which typically exhibit higher pH levels than apoplast fluid (Wilkinson and Davies, 1997). Although direct evidence regarding this transformation is lacking due to limitations on current detection techniques, findings from our *in vitro* experiment may explain morphological changes such as smaller size and roughness observed on shoots and leaves (**Figures 3C, K, L, N**). Nonetheless, the identification of specific factors that trigger the transformation of PLA MPs and determine the resulting products is crucial for future research.

### 3.5 Biological impacts and mechanisms of PLA MPs on plant cells

**Figure 5:**
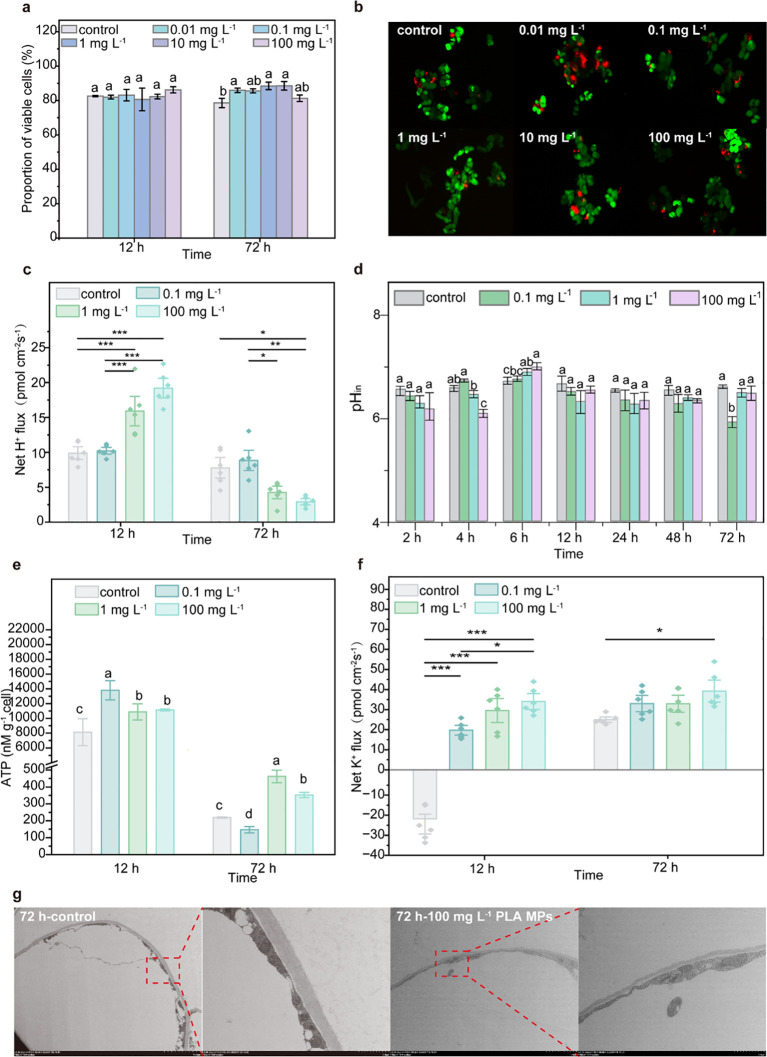
Effects of PLA MPs on cell growth. The viability of cells exposed to 1-100 mg L^-1^ PLA MPs as visualized by FDA/PI staining [n = 5, **(A, B)**]. Dynamic changes in pH_in_ of cells after 72 h exposure of PLA MPs **(C)**. Net H^+^ flux of cells under PLA MP exposure [negative values represent influx, positive values represent efflux, n = 6, **(D)**]. ATP level of cells under PLA MP exposure **(E)**. Net K^+^ flux of cells under PLA MP exposure [negative values represent influx, positive values represent efflux, n = 6, **(F)**]. TEM images of cells under exposure to 100 mg L^-1^ PLA MPs for 72 h **(G)**. Data are represented as means ± SD. ANOVA followed by an LSD test was performed to determine the significant differences within treatments. Statistical ssignificance was considered at *p*<0.05, and the asterisks indicate significant differences between treatments.

Considering the above-mentioned uptake and distribution of PLA MPs in plants, further investigation was conducted to elucidate the impact of PLA MPs on plant cells and their role in promoting plant growth. The viability and fresh/dry weight of cells were evaluated following exposure to PLA MPs. A significant increase (38.5%, *p*<0.05) of cell viability was observed after 72 h of exposure to 100 mg L^−1^ PLA MPs (**Supplementary Figure S10**). In the presence of 1 and 100 mg L^-1^, the fresh weight increased by 48.2-64.1% and 75.8-91.3% for 12 and 72 h exposure, respectively (*p*<0.05, **Supplementary Figure S11**). On this basis, sampling time points at 12 and 72 h were selected for subsequent experiments. The FDA/PI double staining results from cells exposed to PLA MPs for 12 and 72 h demonstrated similar outcomes as those obtained from TTC assay; an increase in cell viability was only observed at 72 h (*e.g.*, by 12.61% for 1 mg L^-1^, *p*<0.05, **Figures 5A, B**; **Supplementary Figure S10**). Consequently, 0.1, 1, and 100 mg L^-1^ were selected as optimal doses for further exploration. Depolymerization of PLA MPs can induce pH variations in the cell culture medium (Liu et al., 2023). Upon the introduction of PLA MPs, a significant reduction in the pH of the culture medium was observed (**Supplementary Figure S12**). This decrease may be due to the depolymerization of PLA MPs, as evidenced by the discovery of an increase in DOC content in the medium after 12 h of cell-free culture (**Supplementary Figure S13**). After short-term exposure (12 h), an increased H^+^ efflux and a rise in pH_in_ occurred concurrently (**Figures 5C, D**). The initial decline in pH_in_ may be due to passive diffusion of H^+^ caused by the decrease in pH_out_ (**Figure 5D**). However, after 72 h, the pH_in_ decreased by 10.3% in the presence of 1 mg L^-1^ PLA MPs (**Figure 5D**, *p*<0.05), which could be attributed to a reduced efflux of H^+^ by an average of 31.5% (**Figure 5C**). Proper regulation of pH is crucial role for plant cell metabolism, signal transduction, and ion transport (Putnam, 2001), and moderate fluctuations in pH can enhance metabolic activity and support cell proliferation (Casey et al., 2010). The increased ATP content further indicates elevated metabolism as a response to PLA MP exposure (**Figure 5E**; **Supplementary Figure S6**). An increase in intracellular ATP content can contribute to active transport of K^+^ (Nguyen et al., 2022). At 12 h, the flow of K^+^ on the cell surface of each treatment changed from influx to efflux, with an increase in the flow rate in a dose-dependent manner (**Figure 5F**). The treatment with 100 mg L^-1^ PLA MPs for 72 h showed the maximum increase in K^+^ efflux flow rate (by 57.2%, **Figure 5F**). This increase in K^+^ efflux indicates an enhanced permeability of the cell membrane (Demidchik et al., 2014), promoting water uptake and cell expansion (Shin, 2017). TEM images revealed that after exposure to 100 mg L^-1^ PLA MPs for 72 h, there was a thinner cell wall (0.56 ± 0.12 μm, n = 12) compared to the control group (0.77 ± 0.19 μm, n = 12, **Figure 5G**), which could be attributed to cell expansion (Cosgrove, 2005). These cellular findings confirmed the promoting effect of PLA MPs observed in maize seedlings. This promotion is most likely attributed to the decrease in extracellular pH resulting from PLA MP transformation and increased K^+^ efflux, which contributed to the cell wall acidification and facilitated cell expansion for plant growth.

## 4 Conclusions

The seed germination and hydroponic experiments demonstrated that low concentrations (1 and 10 mg L^-1^) of PLA MPs increased the germination rate of maize seeds, enhanced shoot height, and increased the dry weight of aboveground biomass. The depolymerization of PLA MPs in the nutrient solution resulted in the formation of small-sized PLA MPs (< 2 μm), with further transformation occurring PLA MPs within the xylem sap and apoplast fluid after 12 h. The growth-promoting effect is mainly attributed to elevated DOC levels due to depolymerization, while exposure to PLA MP stimulates cell wall acidification by mediating H^+^ and increase in K^+^ fluxes. Importantly, our findings provide evidence for the transformation of PLA MPs in hydroponic-plant system; however, further exploration is needed to determine the ultimate products of PLA MPs in mature plants under soil culture conditions. Additionally, advancements in ^13^C isotope labeling techniques may offer greater opportunities for tracking the metabolic pathways of PLA MPs. Moreover, it is important to consider the impact of plastic additives along with biodegradable PLA MPs, particularly their combined effects during the transformation process for a comprehensive understanding of biological effects related to PLA MPs.

## Data Availability

The raw data supporting the conclusions of this article will be made available by the authors, without undue reservation.
