# Supplementary material for: The fate of biodegradable polylactic acid microplastics in maize: impacts on cellular ion fluxes and plant growth
Source: Front Plant Sci. 2025 Feb 25;16:1544298. doi: 10.3389/fpls.2025.1544298 (PMC11893570; doi:10.3389/fpls.2025.1544298)
Supplement: Supplementary file 1 [file DataSheet1.docx]

**Supplementary Information**

**The fate of biodegradable polylactic acid microplastics in maize: impacts on cellular ion fluxes and plant growth**

*Shijia Bao*,^a^ *Xi Wang*,^a^ *Jianxiong Zeng*,^a^ *Le Yue*,^a^ *Zhenggao Xiao*,^a^ *Feiran Chen*,*^,a,b^ *Zhenyu Wang*^a^

^a^ Institute of Environmental Processes and Pollution Control, and School of Environment and Ecology, Jiangnan University, Wuxi 214122, China.

^b^ College of Forestry and Grassland, Nanjing Forestry University, Nanjing 210037, China

*Corresponding author. *E-mail address*: chenfeiran@njfu.edu.cn (Dr. Feiran Chen)

**List** **of** **supplementary information**

**Text S1.** Calculation of indicators related to seed germination.

**Text S2.** Extraction of xylem sap

**Text S3.** Extraction of apoplast fluid

**Text S4.** Measurements of cell viability and fresh/dry weight.

**Text S5.** Measurements of ion fluxes by NMT.

**Figure S1.** Morphology (a) and size distribution (b) of PLA MPs. SEM images and overall size distribution of PLA MPs.

**Figure S2.** Fourier-transform infrared spectroscopy (FTIR) spectra of PLA MPs **(**a). Zeta-potential and hydrodynamic diameter of PLA MPs (b).

**Figure S3.** Xylem sap collection device (a). General extraction procedure of maize leaf apoplast fluid (b).

**Figure S4.** ^1^H (a) and ^13^C (b) NMR spectra of FITC florescence-labeled PLA MPs.

**Figure S5.** Standard curve of intracellular pH.

**Figure S6.** Standard curve of intracellular ATP.

**Figure S7.** Photos of seed germination during a 7-day exposure.

**Figure S8.** Effects of PLA MPs on photosynthesis in hydroponic maize. Chlorophyll content using the SPAD index (a, n=4) and maximum photosystem II quantum yield (Fv/Fm, b, n=6) of maize after 20 d-exposure.

**Figure S9.** Photos of vials for TTC assay (a), and viabilities of BY-2 cells exposed to different doses of PLA MPs (0, 0.01, 0.1, 1, 10, and 100 mg L^-1^, b).

**Figure S10.** Photos of BY-2 cells exposed to different concentrations after 12 and 72 h (a). Fresh or dry weight of BY-2 cells under PLA MPs exposure (b).

**Figure S11.** Dynamic variations in pH of culture medium in the presence or absence of cells.

**Table S1.** Compositions of Hoagland nutrient solution.

**Table S2.** GPC analysis of PLA MPs collected after 20 days of hydroponic incubation in the absence of plants.

**Text S1.** Calculation of indicators related to seed germination

The germination rate (GR), germination vigor (GV), germination index (GI), vigor index (VI) and mean germinating time (MGT) were calculated according to the formula as previously described (Bosker et al., 2019):

$$GR=\frac{n_{7}}{N} \times100\%$$

$$GV=\frac{n_{3}}{N} \times100\%$$

$$GI=\sum\frac{G_{t}}{D_{t}}$$

$$VI=GI \times s$$

$$MGT=\frac{\sum(F\bullet X)}{\sum F}$$

n_7_: the number of seed germinations observed within 7 days.

N: total number of seeds.

n_3_: the number of seed germinations observed within 3 days.

G_t_: number of germinations in t days.

D_t_: the corresponding number of germination days.

s: seedling growth after 7 days (radicle length + bud length).

F: the number of new seed germinations on day X.

X: number of germination days.

**Text S2.** Extraction of xylem sap

The 20-day treated plant was excised approximately 5 cm above the top surface of nutrient solution. The remaining stem was inserted into a flexible plastic tube with an appropriate diameter, containing a steel bead. The joint of the plastic tube with cut end of plant was then sealed with a plastic film, allowing for gentle movement of the steel beads and creating a vacuum between the cut end of the plant and the steel beads within the tube. The xylem sap was exuded through both root pressure and vacuum, which subsequently accumulated in the incision. After one hour, the sap was extracted using an injector and stored at -20°C.

**Text S3.** Extraction of apoplast fluid

Firstly, place the leaf tips in a 60 mL syringe barrel filled with apoplast washing solution (deionized water). Repeated pulling and releasing actions create cycles of vacuum that facilitate penetration of wash solution into the apoplast. Once maximum saturation is achieved, carefully remove leaves from wash solution and gently blot them dry. Next, position these leaves on sealing film measuring 5 × 10 cm and meticulously wrap them around a 1 mL pipette tip to form a structure. This bundle is further secured by another sealing film before being suspended above bottom portion of conical tube (15 mL). Subsequently invert the tube so that leaf tips face downwards and subject it to centrifugation at 2500×g for 10 min at 4°C. Gently resuspend the apoplast liquid together with any solids (*e.g*., bacteria) released from the leaves, transferring this mixture to a 1.5 mL microcentrifuge tube followed by centrifugation for 5 min at 2320×g at 4°C. Finally, transfer the supernatant to a new tube and store it at -20°C.

**Text S4.** Measurements of cell viability and fresh/dry weight

The cell suspension (1 mL) was collected and the medium was removed. Subsequently, the cells were washed three times with phosphate buffer solution (PBS, 0.1 M, pH = 7.2). The cells were resuspended in 1 mL of 0.3% 1,3,5-triphenyltetrazolium chloride (TTC, v/v) dissolved in 50 mM PBS and incubated at a temperature of 25 °C in darkness for 8 h (Dai et al., 2018). Following this, the cells were collected by centrifugation at 6000 rpm to remove PBS and then treated with 95% ethanol. The supernatant was quantified for absorbance at a wavelength of 485 nm using a microplate reader (Thermo Scientific, USA), and technical replications were conducted for quality control. Filtration and washing procedures were performed on the cells before gently squeezing them with tissue paper to eliminate excess liquid for fresh weight (FW) measurement. Finally, the cells were dried at 60 °C for 24 h to determine their dry weight (DW) (Wang et al., 2022).

**Text S5.** Measurements of ion fluxes by NMT

Five mL assay solution (H^+^: 0.1 mM CaCl_2_ + 0.1 mM sucrose, pH = 5.3, K^+^: 0.05 mM KCl, pH = 6.0) was added for equilibration. Pre-pulled and salinized microelectrodes (4.5 ± 0.5 μm, XY-CGQ-01) were filled with a backfilling solution (H^+^: 15 mM NaCl + 40 mM KH_2_PO_4_, pH = 7.0; K^+^: 100 mM KCl) to a length of approximately 1 cm, followed by filling with selective liquid ion-exchange cocktails (LIXs, H^+^: XYSJ-H-10, 50 μm; K^+^: XY-SJ-K-10, 180 μm; Younger, USA). The microelectrodes were calibrated in solutions with 0.1 and 1 mM KCl for K^+^ flux (Nernstian slope 58 ± 5 mV per decade), and with 0.1 mM CaCl_2_ + 0.1 mM sucrose, pH = 5.0 and pH = 6.0 for H^+^ flux (Nernstian slope 58 ± 5 mV per decade) (Wang et al., 2022).

**
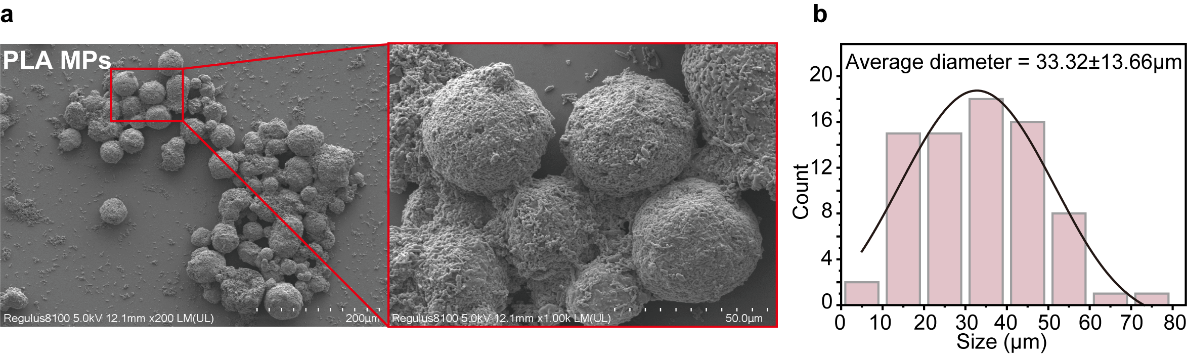
**

**Figure S1.** Morphology **(**a) and size distribution (b) of PLA MPs. SEM images and overall size distribution of PLA MPs. Images are representative of three independent experiments.


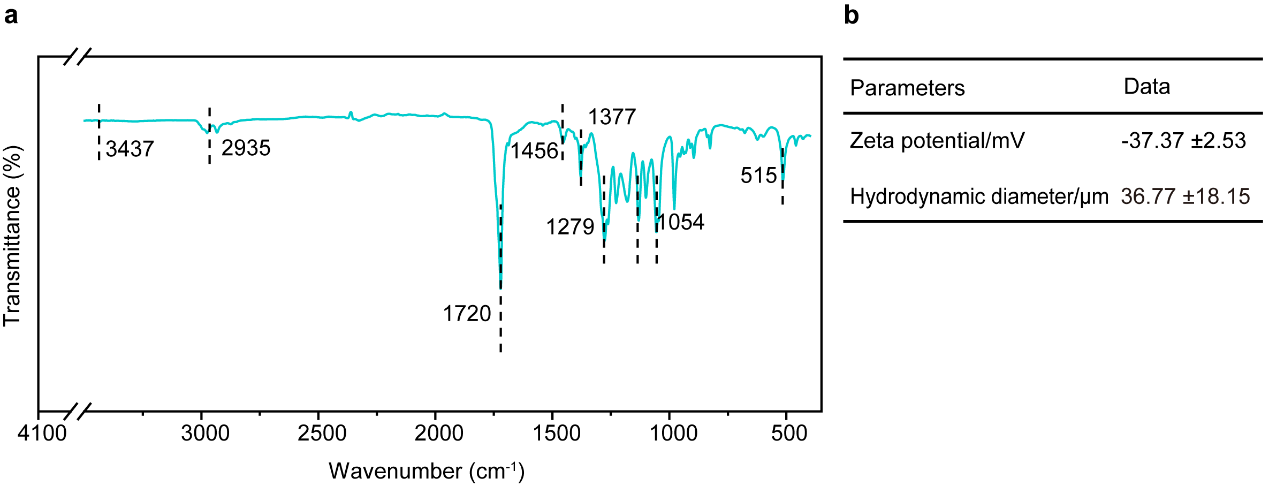


**Figure S2.** Fourier-transform infrared spectroscopy (FTIR) spectra of PLA MPs **(**a). Zeta-potential and hydrodynamic diameter of PLA MPs, data are presented as mean ± SD of three independent experiments (b).

**
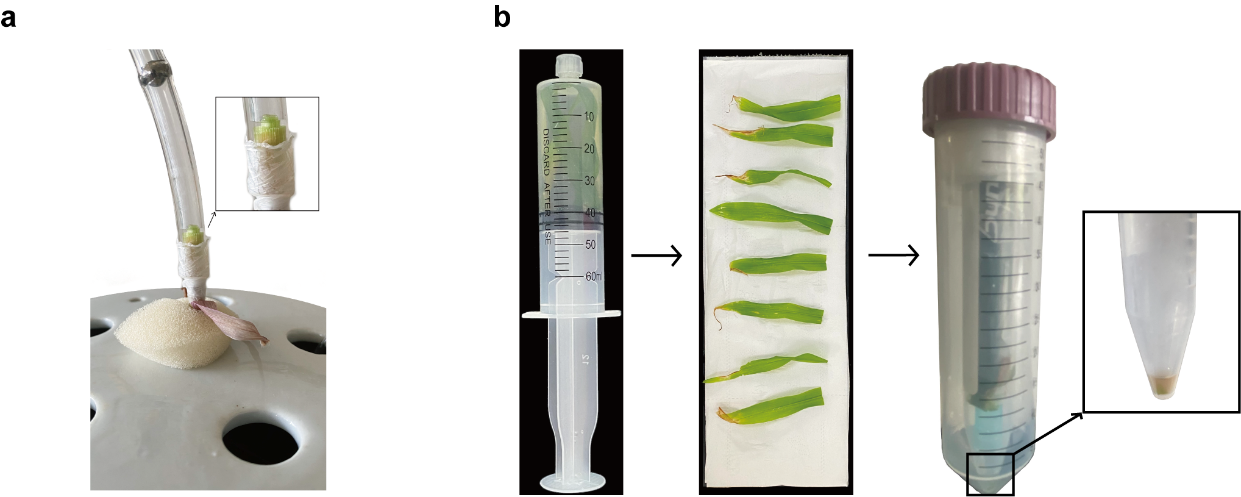
**

**Figure S3.** Xylem sap collection device (a). General extraction procedure of maize leaf apoplast fluid (b).


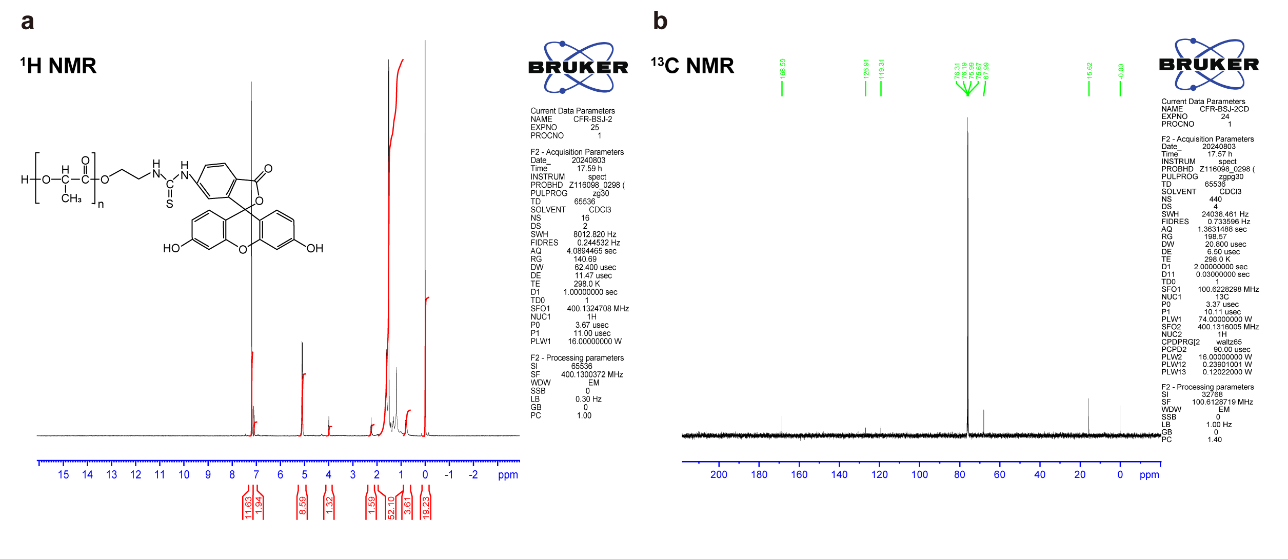


**Figure S4.** ^1^H (a) and ^13^C (b) NMR spectra of FITC florescence-labeled PLA MPs.

**
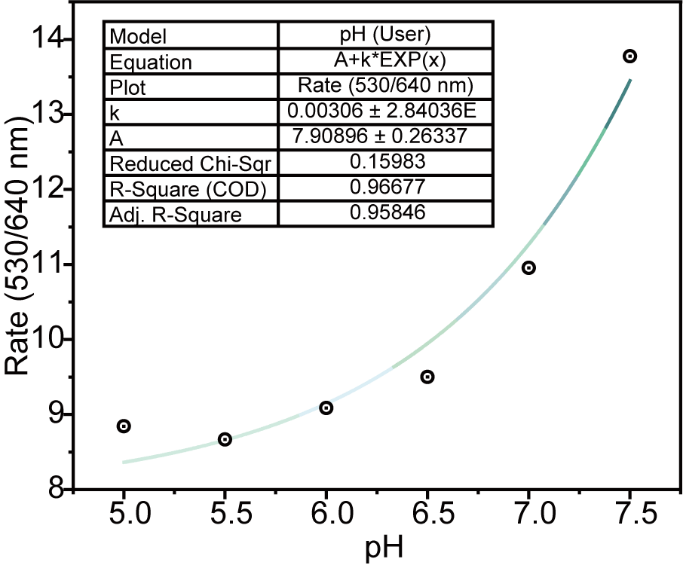
**

**Figure S5.** Standard curve of intracellular pH. Data are presented as mean ± SD of three independent experiments.


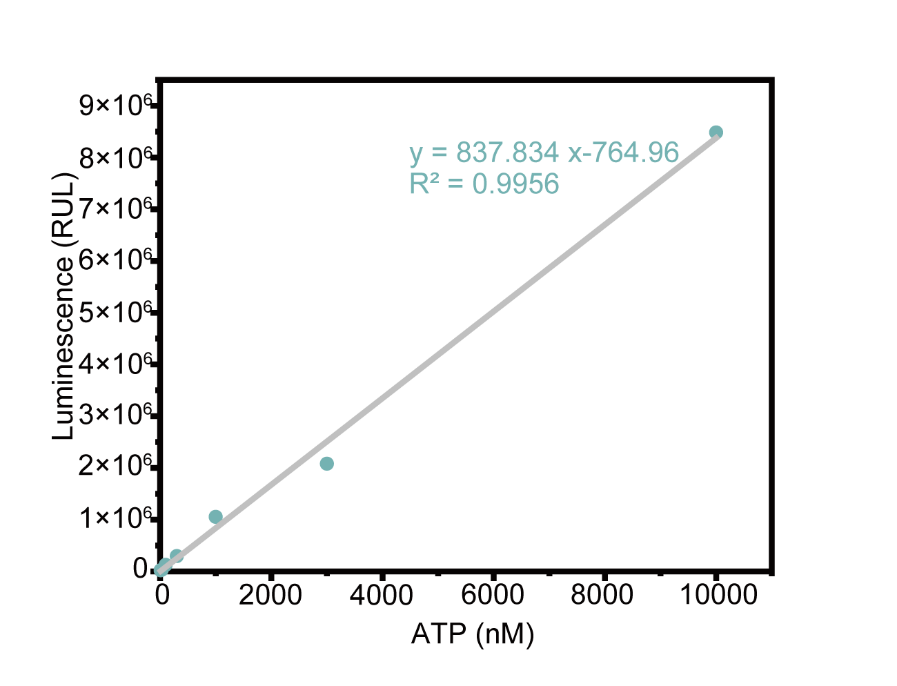


**Figure S6.** Standard curve of intracellular ATP. Data are presented as mean ± SD of three independent experiments.


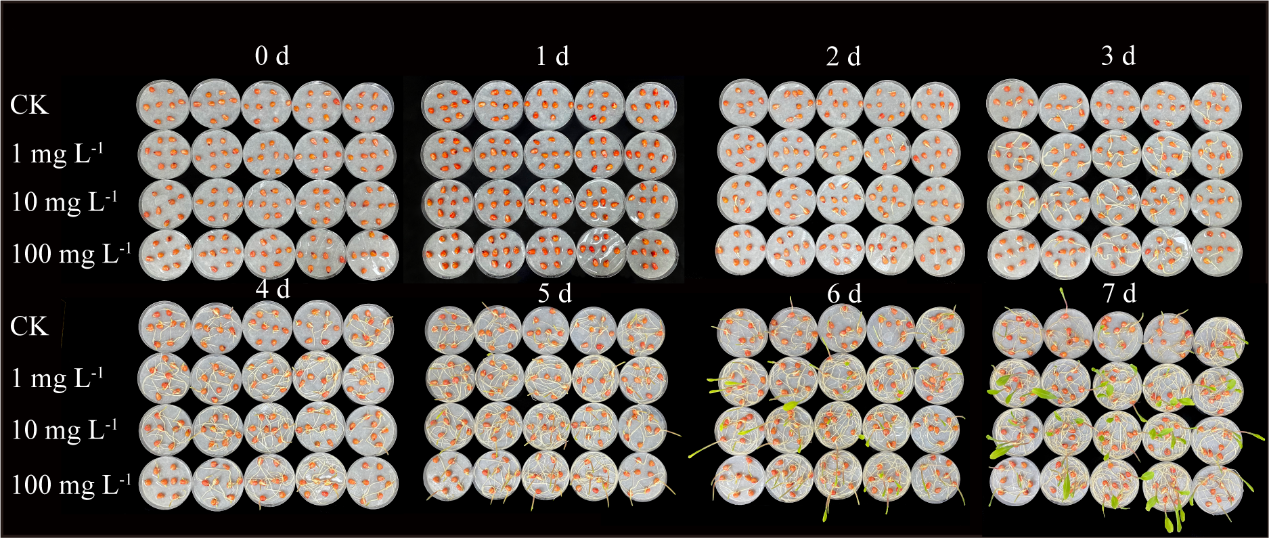
 **Figure S7.** Photos of seed germination during a 7-day exposure.


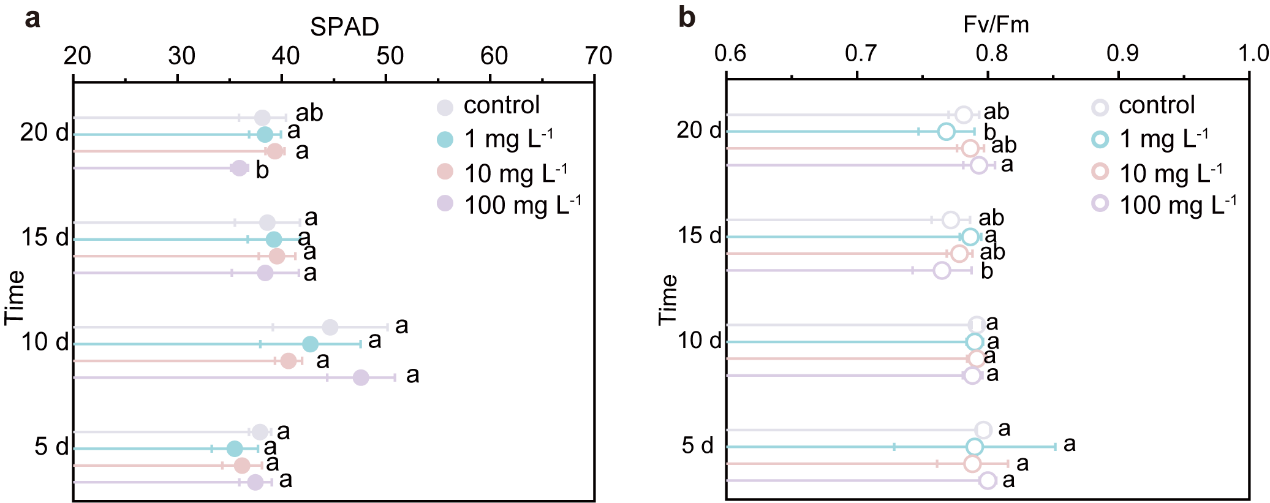


**Figure S8.** Effects of PLA MPs on photosynthesis in hydroponic maize. Chlorophyll content using the SPAD index (a, n = 4) and maximum photosystem II quantum yield (Fv/Fm, b, n = 6) of maize after 20 d-exposure. ANOVA followed by an LSD test was performed to determine the significant differences within treatments. Statistical significance was considered at *p*<0.05.


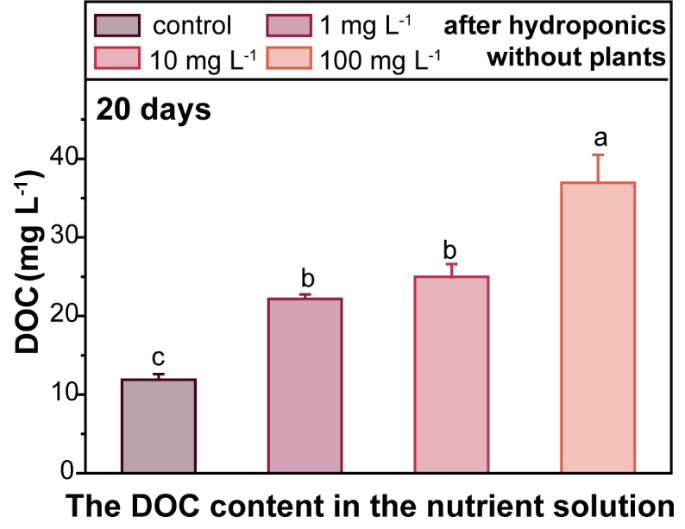


**Figure S9.** The DOC content in the nutrient solution after 20 days of hydroponic culture without plants. Data are represented as means ± SD (n = 3). ANOVA followed by an LSD test was performed to determine the significant differences within treatments. Statistical significance was considered at *p*<0.05.


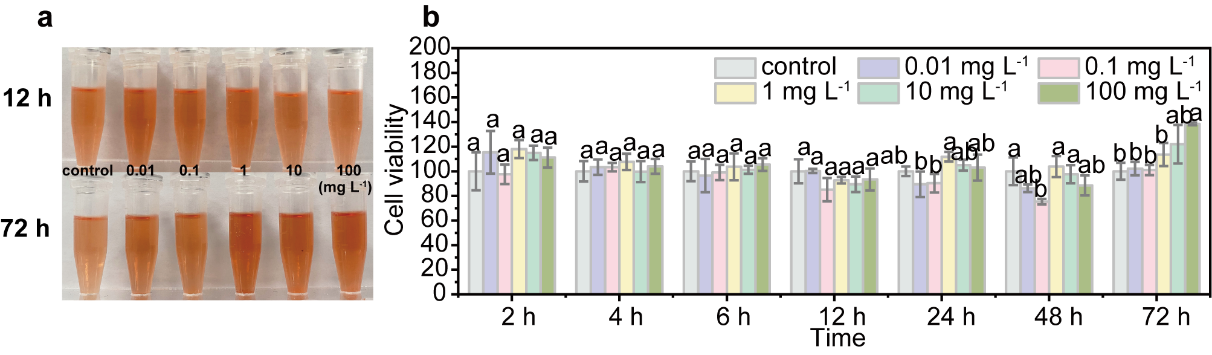


**Figure S10.** Photos of vials for TTC assay (a), and viabilities of BY-2 cells exposed to different doses of PLA MPs (0, 0.01, 0.1, 1, 10, 100 mg L^−1^, b). Data are presented as mean ± SD of three independent experiments (n = 3). ANOVA followed by an LSD test was performed to determine the significant differences within treatments. Statistical significance was considered at *p*<0.05.


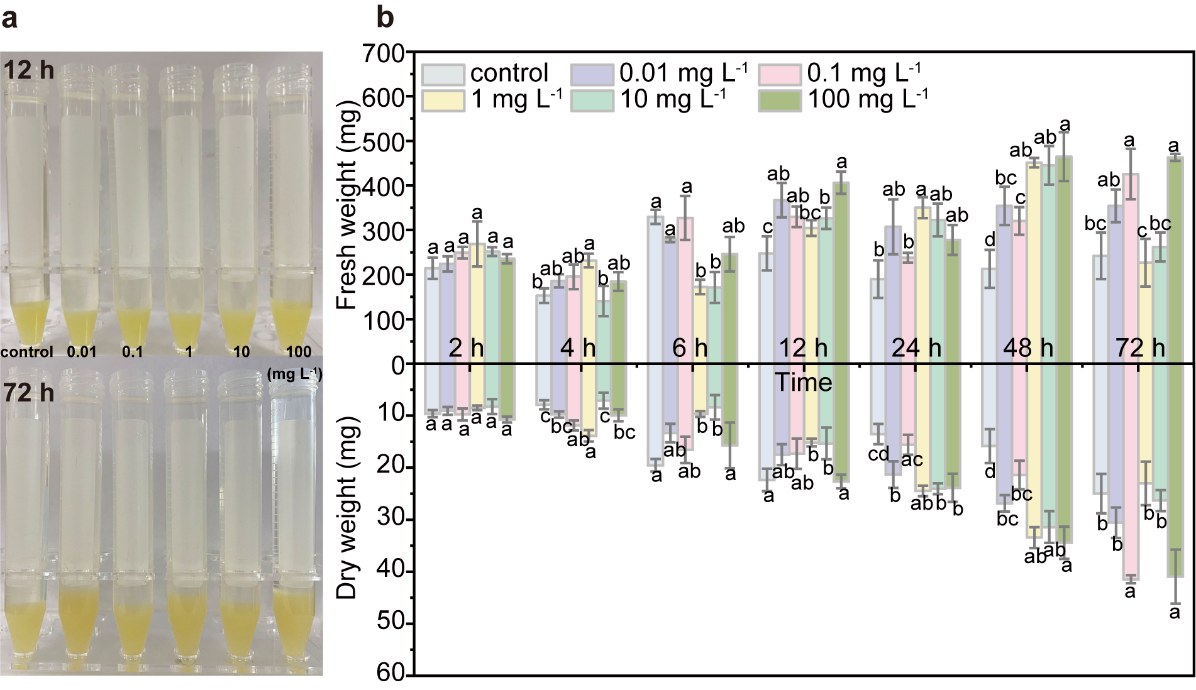


**Figure S11.** Photos of BY-2 cells exposed to different concentrations after 12 and 72 h (a). Fresh or dry weight of BY-2 cells under PLA MPs exposure (b). Data are represented as means ± SD (n = 3). ANOVA followed by an LSD test was performed to determine the significant differences within treatments. Statistical significance was considered at *p*<0.05.


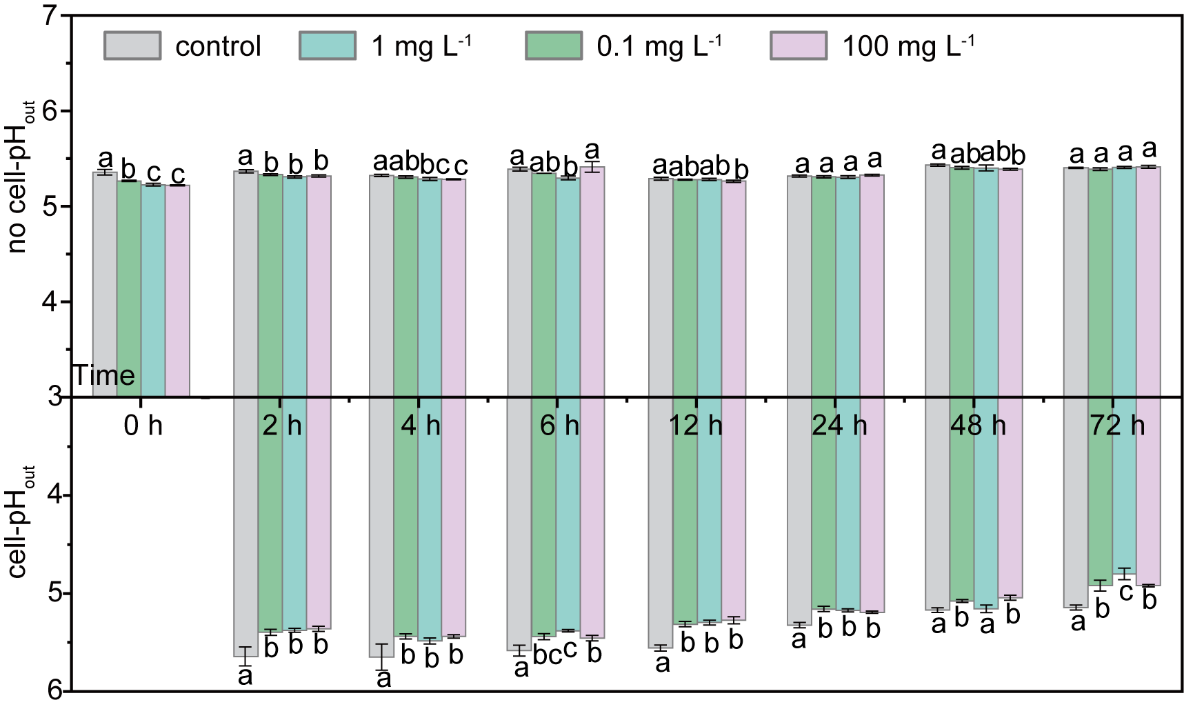


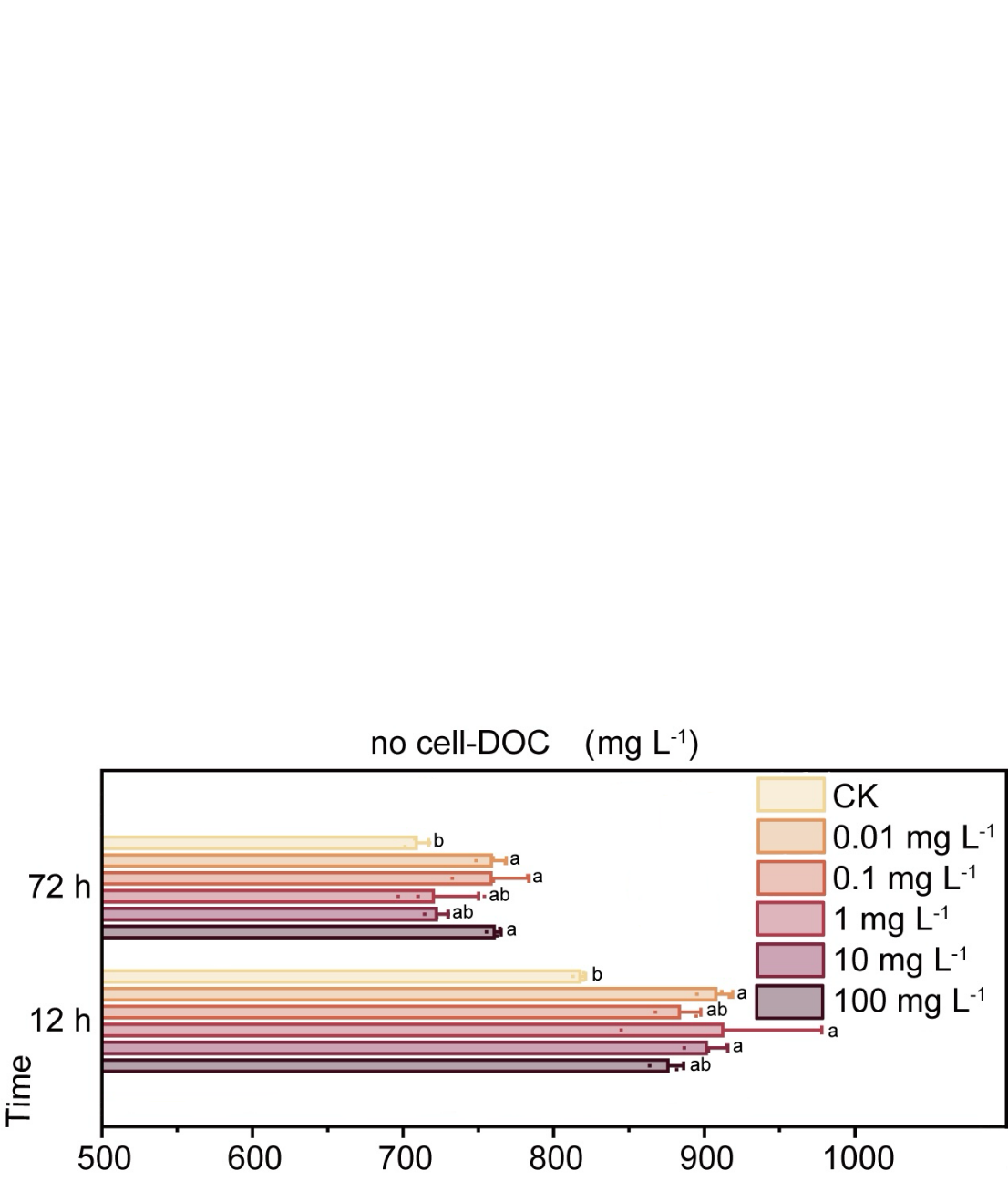
**Figure S12.** Dynamic variations in pH of culture medium in the presence or absence of cells. Data are represented as means ± SD (n = 5). ANOVA followed by an LSD test was performed to determine the significant differences within treatments. Statistical significance was considered at *p*<0.05.

**Figure S13.** The DOC content in the medium after a certain period of culture without BY-2 tobacco cells. Data are represented as means ± SD (n = 3). ANOVA followed by an LSD test was performed to determine the significant differences within treatments. Statistical significance was considered at *p*<0.05.

**Table S1.** Compositions of modified Hoagland nutrient solution.

**Table S2.** GPC analysis of PLA MPs collected after 20 days of hydroponic incubation in the absence of plants.**
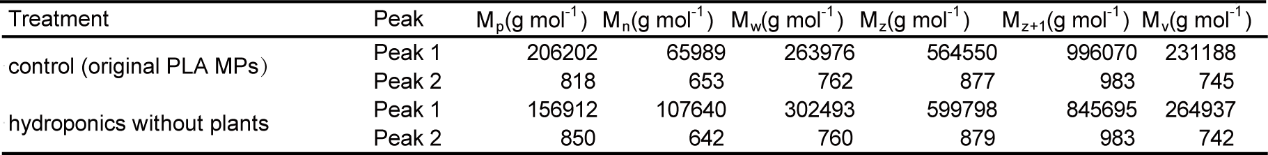
**

**References**

Bosker, T., Bouwman, L. J., Brun, N. R., Behrens, P., & Vijver, M. G. (2019). Microplastics accumulate on pores in seed capsule and delay germination and root growth of the terrestrial vascular plant *Lepidium sativum*. *Chemosphere, 226*, 774-781. doi:https://doi.org/10.1016/j.chemosphere.2019.03.163

Dai, Y., Zhenyu, W., Zhao, J., Xu, L., Xu, L., Yu, X., Wei, Y., & Xing, B. (2018). Interaction of CuO nanoparticles with plant cells: Internalization, oxidative stress, electron transport chain disruption, and toxicogenomic responses. *Environmental Science: Nano, 5*. doi:https://doi.org/10.1039/C8EN00222C

Wang, Z., Tang, J., Zhu, L., Feng, Y., Yue, L., Wang, C., Xiao, Z., & Chen, F. (2022). Nanomaterial-induced modulation of hormonal pathways enhances plant cell growth. *Environmental Science: Nano, 9*(5), 1578-1590. doi:https://doi.org/10.1039/D2EN00251E
